# Study on the Correlation between Gene Expression and Enzyme Activity of Seven Key Enzymes and Ginsenoside Content in Ginseng in Over Time in Ji’an, China

**DOI:** 10.3390/ijms18122682

**Published:** 2017-12-11

**Authors:** Juxin Yin, Daihui Zhang, Jianjian Zhuang, Yi Huang, Ying Mu, Shaowu Lv

**Affiliations:** 1 Key Laboratory for Molecular Enzymology and Engineering of the Ministry of Education, College of Life Science, Jilin University, Changchun 130000, China; yinjuxin@163.com (J.Y.); zhuangjj15@mails.jlu.edu.cn (J.Z.); huangyimzd@126.com (Y.H.); 2 Research Center for Analytical Instrumentation, Institute of Cyber-Systems and Control, State Key Laboratory of Industrial Control Technology, Zhejiang University, Hangzhou 310000, China; muying@zju.edu.cn; 3 Jilin Entry Exit Inspection and Quarantine Bureau, Changchun 130000, China; Zhangdaihui1235@163.com

**Keywords:** fresh ginseng, ginsenoside, key enzyme, enzyme activity, gene expression, correlation

## Abstract

*Panax ginseng* is a traditional medicine. Fresh ginseng is one of the most important industries related to ginseng development, and fresh ginseng of varying ages has different medicinal properties. Previous research has not systematically reported the correlation between changes in key enzyme activity with changes in ginsenoside content in fresh ginseng over time. In this study, for the first time, we use ginseng samples of varying ages in Ji’an and systematically reported the changes in the activity of seven key enzymes (HMGR, FPS, SS, SE, DS, CYP450, and GT). We investigated the content of ginsenoside and gene expression of these key enzymes. Ginsenoside content was measured using HPLC. HPLC, GC-MS, and LC-MS were combined to measure the enzyme activity of the key enzymes. Quantitative PCR was used in the investigation of gene expression. By analyzing the correlation between the enzyme activity and the transcription level of the key enzymes with ginsenoside content, we found that DS and GT enzyme activities are significantly correlated with the ginsenoside content in different ages of ginseng. Our findings might provide a new strategy to discriminate between ginseng of different years. Meanwhile, this research provides important information for the in-depth study of ginsenoside biosynthesis.

## 1. Introduction

The root of Asian ginseng (*Panax ginseng* Meyer), which has been used as an important herbal medicine, has existed for thousands of years in China, Japan, and Korea [1]. The root has crucial nutritive value, is particularly cultivated in Chinese traditional culture, and the reputation of its effectiveness has reached mythic proportions [2,3]. Modern medicine has evidenced that the most important active ingredient in *ginseng* is ginsenoside, which has significant benefits for humans including anticancer [4,5], immunomodulatory, and anti-diabetic properties [6,7], among others. The ginsenosides can be classified into two groups (dammarane-type and oleanane-type) by the structure of the aglycones. The dammarane ginsenosides are the primary type of ginsenosides, and they consist of protopanaxadiol (PPD)-type saponins and protopanaxatriol (PPT)-type saponins [2].

**Figure 1:**
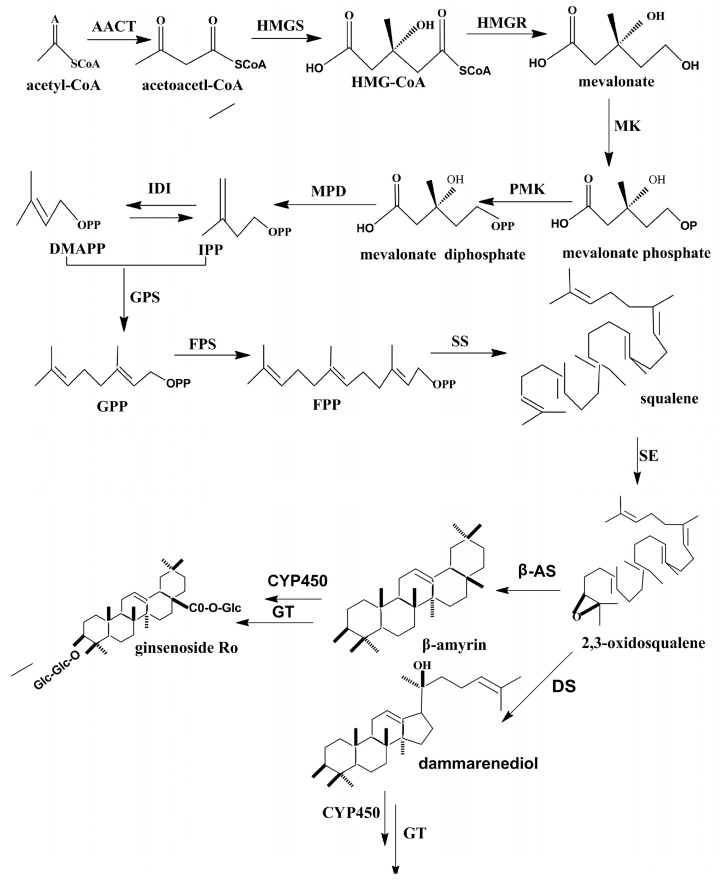
The biosynthesis pathway of dammarane ginsenoside. (AACT: acetyl-CoA C-acetyltransferase; HMGS: hydroxy methyl glutaryl-CoA synthase; IDI: isopentenyl pyrophosphate isomerase; MPD: mevalonate pyrophosphate decarboxylase; PMK: Phosphomevalonate kinase; MK: mevalonate kinase; GPS: Geranyl Pyrophosphate Synthase: β-AS: β-amyrin synthase).

The ginsenoside biosynthesis pathway of the dammarane-type has been reported by our groups [8,9,10]. As shown in Figure 1, the biosynthesis of ginsenoside is mainly completed through three reaction steps. In the first step, isopentenyl diphosphate (IPP) and its isomer dimethylallyl diphosphate (DMAPP) are generated through several reactions, and the key enzyme is 3-hydroxy-3-methylglutaryl CoA reductase (HMGR). The above is the first stage. Farnesyl diphosphate (FPP) is produced by the catalysis of farnesyl diphosphate synthase (FPS). As a substrate, FPP generates squalene by the catalysis of squalene synthase (SS) [9]. Squalene epoxidase (SE) acts as the substrate in the formation of 2,3-oxidosqualene. The 2,3-oxidosqualene can be converted into protopanaxadiol; this conversion is dependent on dammarenediol synthase (DS). Cytochrome P450 (CYP450), which is involved in modifications of the ring and side chain in terpene biochemistry, can convert protopanaxadiol to protopanaxatriol. Finally, different amounts of monosaccharides are added to the triterpene aglycones by glycosyltransferase (GT), and the procedure will lead to the generation of different ginsenosides.

As an important medical herb, fresh ginseng is an important economic industry in northeastern China [11,12]. Ginsenoside has substantial medicinal value, and the Chinese pharmacopeia clearly stipulates that the ginsenoside content must reach a certain degree before it can be used as a medicine. The roots of fresh ginseng have more malonyl ginsenosides, amino acids, and polysaccharides than dried roots [13,14]. Meanwhile, fresh ginseng may have better medicinal effects and an overall higher quality than wild ginseng or other products [13,15]. The ginsenoside accumulates over time, and fresh ginseng, therefore, varies in its medicinal effects based on age [16]. Liu et al. has reported the correlation of ginsenoside with the expression of key genes in ginseng with different cultivation times [17].

However, there has been no reports on the correlation between the ginsenosides and key enzyme activity in the biosynthesis of ginsenosides. Additionally, there have been no reports of the seven key enzymes examined in our study in the synthesis of ginsenosides. We used fresh ginseng roots of different ages. In addition to systematically measuring the activity of seven key enzymes in the synthesis of ginseng saponins in fresh ginseng for the first time, we also measured the gene expression of the same seven key enzymes. We aimed to examine the relationship between the changes in the activity of these key enzymes and gene expression with the ginsenoside content in ginseng of varying ages. The results showed that DS and GT enzyme activity are significantly correlated with the ginsenoside content in ginseng. Here, we propose a new strategy to identify different years of fresh ginseng, and this strategy can provide a reference for identifying the age of ginseng.

## 2. Results

### 2.1. The Content of Total Saponin

**Figure 2:**
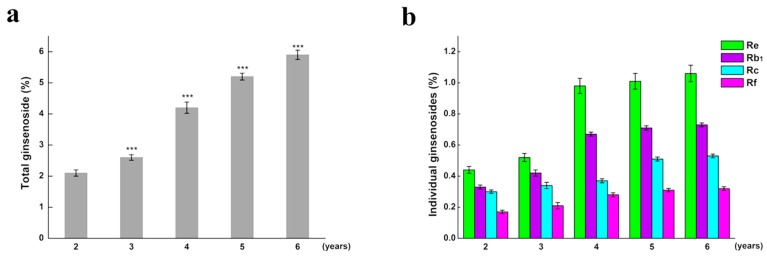
The total and monomer ginsenosides contents at different ages of fresh *P. ginseng*. (**a**) Total ginsenosides content; (**b**) Monomer ginsenosides content. (Tukey’s multiple comparison test, *** *p* < 0.001 vs. the previous year; *n* = 60).

**Figure 3:**
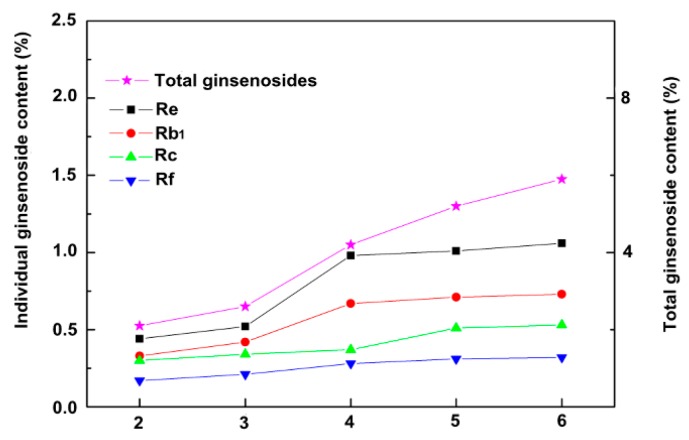
The changes of total and individual ginsenoside contents at different ages of fresh ginseng growth. The left *Y*-axis represents individual ginsenoside contents, while the right side of the *Y*-axis represents total ginsenoside content.

As growth increased over the years, the total saponins content accumulated over time. Compared to the previous year, total saponins significantly changed in year 3. We compared the changes of monomer saponins and total saponins in different years (Figure 2). We observed that the total saponins content of ginseng increased from years 2–6, during which the total content of saponins increased from years 3–4 more than that in the other years. Individual ginsenosides were quantified using HPLC. The amount of monomer saponins in ginseng also increased annually, and the increase of monomer saponins content from years 3–4 was higher than that in other years (Figure 3). The retention times of individual ginsenoside were: Re: 65.19 min; Rf: 85.23 min; Rb1: 90.22 min, and Rc: 97.10 min (Figure S1).

### 2.2. The Expression of Key Enzymes

**Figure 4:**
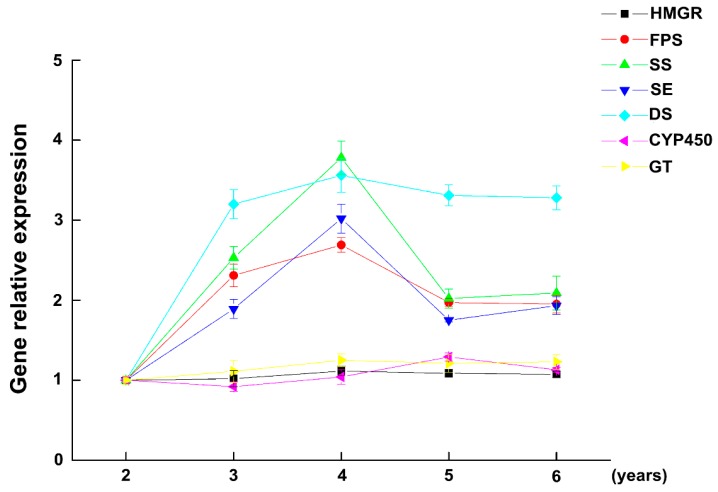
The changes in gene expression in fresh ginseng roots after different years of growth. (Gene expression at two years of growth was the control group, *n* = 60 ginseng).

Gene expression levels of *HMGR*, *FPS*, *SS*, *SE*, DS, *CYP450*, and *GT* in fresh ginseng were analyzed using qRT-PCR. Gene expression after two years of growth was the control group and the changes of expression are shown in Figure 4. The expression level of most genes peaked in the fourth year, indicating that it is the same year when growth peaks. This observation is consistent with total saponin changes from years 3–4. The increase in *FPS*, *SS*, and *SE* gene expression levels between the second and fourth year is possibly because 2,3-squalene is a common precursor of sterol and triterpenoids, and the *FPS*, *SS*, and *SE* genes are expressed as enzymes in the role of using 2,3-oxidosqualene to meet the needs of growth and development. The expression of *FPS*, *SS*, and *SE* decreased from the fourth to sixth years, indicating that the accumulation of 2,3-squalene was then sufficient to meet the requirements for synthesized substances. A sufficient supply of 2,3-oxidosqualene is important, as it is a basic precursor for ginsenoside synthesis. *HMGR* is stable over several years, likely because it is a rate-limiting enzyme in the main pathway of IPP and DMAPP synthesis [18]. *HMGR* needs to be kept at a relatively high level of expression to meet the requirements for growth and development. The expression of the *DS* gene went through a rapid increase from the second through fourth years and then stabilized. DS is directly related to the synthesis of ginsenoside precursor, and its expression rapidly increased during the second to fourth years. This observation is consistent with the changes in total saponins at this stage. Subsequently, *DS* expression stabilized, indicating that the synthetic rates of saponins were also stable. CYP450 and GT have a direct relationship with ginsenoside synthesis. The expression of the genes is at a relatively stable level over several years. The possible reasons are that the synthesis of ginsenosides is a continuous metabolic activity over several years, the tricyclic triterpene system is subject to continuous hydroxylation and glycosylation, and therefore, *GT* and *CYP450* genes are required to synthesize ginsenosides at a relatively stable level. 

### 2.3. The Activity of Key Enzymes

**Figure 5:**
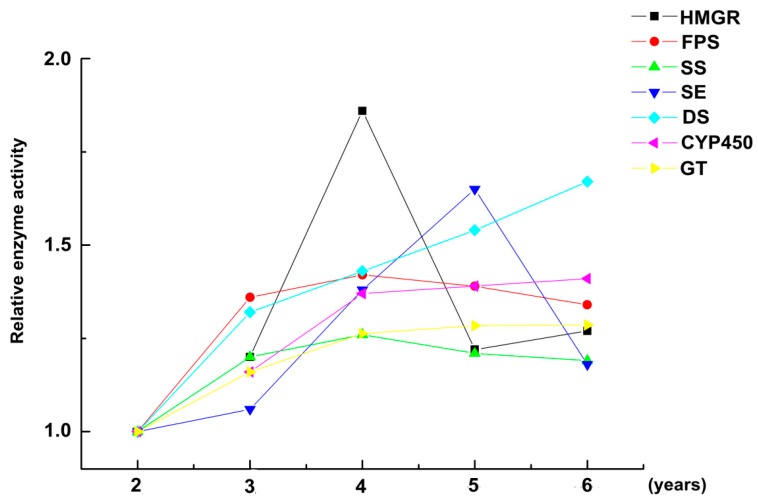
The changes in relative enzyme activity in fresh ginseng roots after different years of growth. (Enzyme activity at two years of growth was the control group, *n* = 60 ginseng).

Different methods were used to measure the activities of key enzymes. The enzyme activity of ginseng grown for two years was used as the control group. The activities of four key enzymes (HMGR, FPS, SS, and SE) increased over time from the second to fourth years, reaching their highest activity in the fourth year (Figure 5). The activities subsequently gradually decreased. However, the activities of the three remaining key enzymes (DS, CYP450, and GT) continuously increased from the second to the sixth years. This trend was consistent with total saponins across different years. 

### 2.4. Correlation Analysis

**Figure 6:**
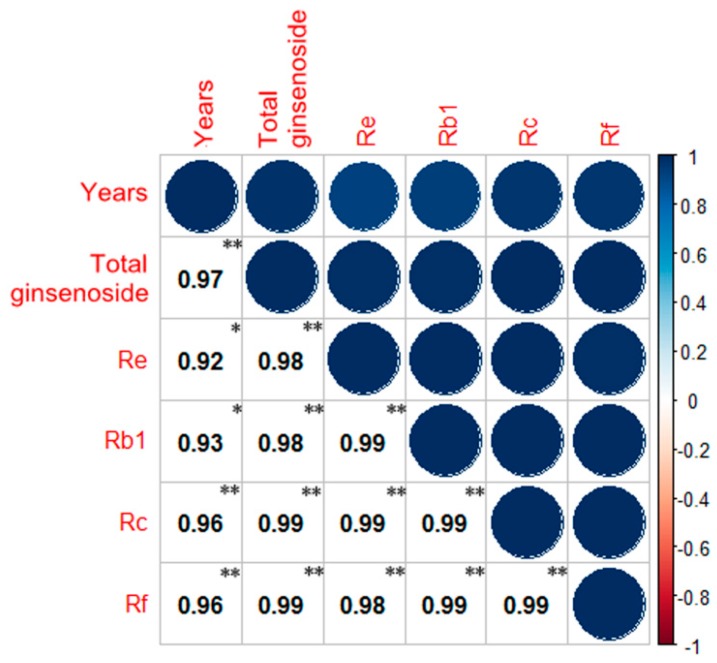
The correlation analysis of total ginsenoside and individual ginsenosides. (** The correlation is significant, with a confidence level (double test) of 0.01. * The correlation is significant, with a confidence level (double test) of 0.05.)

The correlation of total saponins and monomer saponins in fresh ginseng was analyzed using SPSS Software and R (Figure 6). A strong correlation is apparent between the monomer saponins and total saponins. The level of confidence of monomer saponins and total saponins in the different years (2–6 years) of fresh ginseng growth is less than 0.01, indicating a significant correlation. This might be because of the fact that monomer saponins and total saponins share a common metabolic pathway.

**Figure 7:**
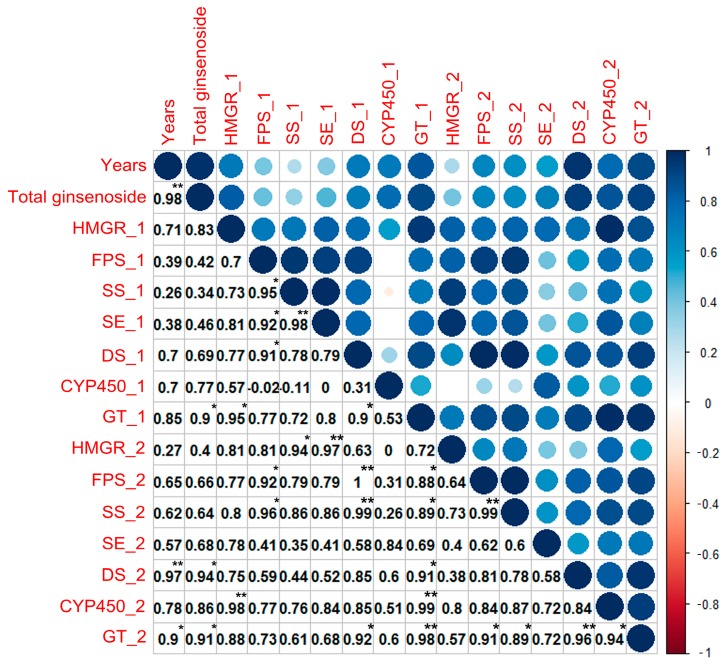
The correlation analysis of total ginsenoside and the individual ginsenosides. (** The correlation is significant, with a confidence level (double test) of 0.01. * The correlation is significant, with a confidence level (double test) of 0.05. Underscore 1 (_1) represents the gene expression of a key enzyme. Underscore 2 (_2) represents the activity of a key enzyme).

The correlation of total ginsenoside with the gene expression and enzyme activities of key enzymes is shown in Figure 7. The order of the correlation between the expression of the key enzymes and years was HMGR (0.71), DS (0.70), P450 (0.70), FPS (0.39), SE (0.38), and SS (0.26). The correlation between the expression of other key enzymes and years was strongly correlated for GT, HMGR, DS, and CYP450; however, because the level of confidence was greater than 0.05, these correlations are not significant. The order of the correlation between the enzyme activity of key enzymes and years was DS (0.97), GT (0.90), P450 (0.78), FPS (0.65), SS (0.62), SE (0.57), and HMGR (0.27). Only DS and GT had a level of confidence less than 0.05, so only the relationships between GT and DS enzyme activity and year are significant. The correlation between the expression of key enzymes and total saponins in the different years (2–6 years) was GT (0.90), HMGR (0.83), CYP450 (0.77), DS (0.69), SE (0.46), FPS (0.42), and SS (0.34). However, their gene expression did not demonstrate significant correlations with total saponins content. The correlation between the activity of key enzymes and the total saponin per year was DS (0.94), GT (0.91), P450 (0.86), SE (0.68), FPS (0.66), SS (0.64), and HMGR (0.40). Only GT and DS had confidence levels less than 0.05, so the enzyme activity of both GT and DS and total saponins are significantly correlated. 

## 3. Discussion

Fresh ginseng was harvested in October each year, and the development of modified atmospheres packing (MAP) enables research on fresh ginseng [14,19,20,21,22]. Ginseng is an important traditional folk medicine in China, Japan, and Korea [5], and its medical effect may be better than wild ginseng [15]. The age of ginseng root is essential to the development of the ginseng industry. Our results have provided correlations between the activity of key enzymes and ginsenoside content in ginseng of different years in Ji’an, China. The results might yield insight into a new strategy for identifying the age of individual ginseng plants. Several studies have reported other methods to discriminate the different ages of ginseng, such as telomere length [23], allometry [24] Fourier transform infrared spectroscopy (FTIR) [25], and taste-sensing [26].

To the best of our knowledge, we systematically report for the first time, the change in the activity of seven key enzymes in fresh ginseng. Through correlation analysis, we propose a potential new strategy for discriminating the age of fresh ginseng roots by measuring the content of ginsenoside, gene expression, and enzyme activity. First, we successfully measured total saponins and developed an HPLC-based method for the simultaneous determination of four types of individual ginsenosides. Moreover, the HPLC method presented here was a rapid and sensitive way to quantify the content of major individual ginsenosides in fresh ginseng samples [17]. The observed trend in the change and content of total saponins and monomer saponins is consistent with previous research [27,28,29]. The correlation of total ginsenosides and individual ginsenosides had statistical significance and obviously increased with age. Their common metabolic pathway results in a correlation between saponins and ginsenosides. To further understand the molecular mechanisms of ginsenoside biosynthesis at different ages, we determined the transcription level of seven key genes (*HMGR*, *FPS*, *SS*, *SE*, *DS*, *CYP450*, and *GT*) in the ginsenoside biosynthetic pathway. Gene expression exhibited different variation trends for different phases of growth. Gene expression during the first stage (*HMGR*) and the third stage (*GT*, *CYP450*) had a similar tendency across the different ages. The two enzymes are required throughout all stages of ginsenoside biosynthesis [30]. The genes encoding the three key enzymes presented the same trend during the second biosynthesis stage, and the trend was similar to results of the present study [17]. *DS* gene expression rapidly increased from years 2–4 and then remained stable during years 4–6; these results were consistent with changes in ginsenoside. The *DS* gene is relevant to dammarenediol, which is the direct precursor of the dammarane tetracyclic triterpenoids. However, the correlation of gene expression with total saponins and years did not reach statistical significance.

Key enzyme activity was measured, and the data were normalized for processing. The activity for all seven enzymes significantly increased between the second and fourth years, indicating a rapid increase of ginsenoside during this period. This phenomenon was consistent with the change in ginsenoside. The correlation was analyzed, and only DS and GT activities were statistically significant. Previous studies reported that the initial committed step in ginsenoside synthesis is the transformation of 2,3-oxidosqualene to dammarenediol II catalyzed by DS, which is the first dedicated enzyme for ginsenoside biosynthesis in *P. ginseng*. Several studies have shown that the up-regulation of DS expression resulted in increased ginsenoside. The enzyme activity of DS could directly control the biosynthesis of ginsenoside [8,31,32]. GT is a critical enzyme that is related to the generation of protopanaxadiol and protopanaxatriol and has important regulatory effects. Glycosylation is an important mechanism for protein function modification. Glycosylation is generally the last step in the biosynthesis of secondary metabolites [33]. In ginseng, GT could convert the aglycons (protopanaxadiol, protopanaxatriol, and oleanolic acid) into ginsenoside compounds [34]. Ginsenosides (such as Re and Rf) have different biological and pharmacological effects, and GT plays a crucial role in the modification of protopanaxadiol, protopanaxatriol, and oleanolic acid [35].

Previous research has largely ignored the activity of key enzymes in ginseng. There is a significant relationship between ginsenoside content and enzyme activity. In this study, we systematically reported a detection method for enzyme activity in fresh ginseng. From the correlation analysis for the material of Ji’an, combined with changes in the enzyme activity of DS and GT across several years, we conclude that the DS and GT enzyme activity in different years is of great significance. Therefore, the results may provide a new strategy to determine the age of fresh ginseng. We have additionally provided a basic experimental dataset for further studies on key enzyme activity in ginseng.

## 4. Methods

### 4.1. Plant Material

Fresh ginseng roots were collected from 60 plants of different ages (2–6 year) in Ji’an, China in October 2015 and 2016. The fresh material was stored using modified atmosphere packing (MAP) [19].

### 4.2. Extraction of Ginseng Total Saponins

One gram of fresh root material was mashed using a cryogenic grinding machine (Shanghai Jing Xin Industrial Development Co., Ltd., Shanghai, China) and placed into centrifuge tubes. Ten milliliters of 70% ethanol was added to the mashed roots, and the samples were shaken at a speed of 100 r/min overnight. Samples were treated three times with ultrasonic waves (40 KHz) for 30 min. The extracted solutions were merged for rotary evaporation until no liquid residue remained. Distilled water (10 mL) and ether (10 mL) were added into the separatory funnel, repeated three times, with the water layer recovered. The samples were extracted an additional three times by water-saturated butanol (10 mL). Distilled water (10 mL) was added to remove sugar three times, recovering the organic layer. The solution was merged for rotary evaporation until no liquid residue remained. A chromatographically pure methanol solution (10 mL) was added and stored in a brown bottle to avoid light damage.

### 4.3. Analysis of Ginsenosides by HPLC

The samples were dissolved in methanol and then filtered through a 0.45 μm filter for HPLC analysis. The HPLC separation was performed on an HPLC-20A system (Shimadzu, Kyoto, Japan) equipped with an autosampler and a UV detector using a C18 column (250 mm × 4.6 mm, 5 μm; SUPELCO, Milwaukee, WI, USA). The mobile phase was solvent A (100% acetonitrile) and solvent B (100% water). The gradient program was: 0–32 min, 18% A (isocratic); 32–50 min, 18–25% A; 50–70 min, 25–30% A; 70–100 min, 30–35%, and 100–120 min, 35–70%. The sample injection volume was 10 µL, and the temperature was 25 °C. The flow rate was 1.0 mL/min, and UV absorption was measured at 203 nm. Quantitative analysis was performed using the standard curve method with authentic ginsenoside as the external standard (Sigma-Aldrich, St. Louis, MO, USA).

### 4.4. Quantification of Transcript Levels

One-hundred milligrams of fresh ginseng roots were mashed with a cryogenic grinding machine at a low temperature. Total RNA was extracted from the ginseng with the MiniBEST Universal RNA Extraction kit (Takara, Tokyo, Japan), including the DNase I digestion step. Next, total RNA was reverse-transcribed using the PrintScript RT Reagent kit with a gDNA Eraser (Takara). A real-time quantitative polymerase chain reaction was performed using 1 µg cDNA in a reaction volume of 20 µL using SYBR Premix Ex Taq™ (Takara). The thermal cycler conditions recommended by the manufacturer were used: 10 min at 95 °C, followed by 40 cycles at 95 °C for 3 s, 60 °C for 34 s. The fluorescent product was detected during the final step of each cycle. Amplification, detection, and data analysis were carried out on an ABI 7500 real-time rotary analyzer. The primers used are presented in Table S1. To determine the relative fold-differences in template abundance for each sample, the *C*_t_ values for each of the gene-specific primers were normalized to the *C*_t_ value for *GAPDH* (5′-GGTGTAACCTAAGATTCCCTTGAGT-3′ and 5′-ACTGTCAGGTTGGCGAAGAAG-3′) and calculated relative to a calibrator using the formula 2^−^^△△*C*t^. 

### 4.5. Enzyme Activity Assay

#### 4.5.1. Protein Extraction

The protein was extracted by a Plant Total Protein extraction kit (Bestbio, Shanghai, China). Ginseng hairy roots were ground with the cryogenic grinding machine and kept at 4 °C. Total protein was determined using the BCA Protein quantitation kit (Bestbio).

#### 4.5.2. HMGR Activity Assay

The activity assay of HMGR was measured by the HMG-CoA Reductase Assay Kit (Sigma-Aldrich). The definition of enzyme activity unit: one unit will convert 1.0 µmol of NADPH to NADP^+^ per 1 min at 37 °C. The unit-specific activity is defined as µmol/min/mg protein (units/mg protein).

#### 4.5.3. FPS Activity Assay

The reaction was performed according to [36], with certain degree changes. IPP (80 μM) and DMAPP (40 μM) were incubated with crude enzyme (20 μg) in 200 μL of buffer (50 mM Tris-HCl, pH 7.0; 50 mM MgCl_2_, and 2 mM DL-1,4-Dithiothreitol). The reactions were conducted at 37 °C for 30 min, and samples were further incubated overnight in the presence of 20 U shrimp alkaline phosphatase (Sigma-Aldrich) to hydrolyze GPP and FPP into their extractable forms, geraniol and farnesol. The sample was extracted with 200 μL hexane on ice. The hexane extract was dried over anhydrous Na_2_SO_4_ and analyzed using GC-MS (Agilent, Palo Alto, CA, USA). The system was equipped with a non-polar DB-5 capillary column (30 m × 0.25 mm × 250 µm), and the carrier gas was helium (1.0 mL/min). The column temperature started at 60 °C and increased at a rate of 8 °C/min until 270 °C; it was held at 305 °C for 3 min. The mass spectrometer was operated in the electron impact ionization mode (EI, 70 eV) and the scan mode in the range of 20–600 *m*/*z*. Different concentrations of farnesol (Sigma-Aldrich) were also prepared for the standard curve and analyzed by GC-MS in the same conditions. 

#### 4.5.4. SS Enzyme Activity

The reaction mixture contained 100 mM Tris-HCl (pH 7.5), 10 mM farnesyl pyrophosphate triammonium salt (Sigma-Aldrich), 10 mM MgCl_2_, 1 mM DTT, 2% glycine, 3 mM NADPH, 20 μg crude enzyme, and the total volume was 1 mL. The reaction was performed at 32 °C for 10 h and then extracted with 200 µL hexane [37]. The analysis was performed on a GC-MS system (Agilent), which was equipped with a DB-5 column (30 m × 0.25 mm × 250 µm) and the carrier gas was helium (1.0 mL/ min). The column temperature was maintained at 120 °C for 3 min, at the rate of 15 °C/min to 180 °C, then elevated to 260 °C at 25 °C/min, and finally held at 305 °C for 3 min. The mass range was 20–600 *m*/*z* and performed in scan mode. Different concentrations of squalene (Sigma-Aldrich) were also prepared for the standard curve and analyzed by GC-MS using the same conditions as described above.

#### 4.5.5. SE Enzyme Activity

The Plant Squalene epoxidase ELISA Kit (TSZ, Framingham, MA, USA) was used to measure the activity of SE. The concentration of SE in the samples was then determined by comparing the optical density (OD) of the samples to the standard curve.

#### 4.5.6. DS Enzyme Activity

The reaction system, a total volume of 500 µL, contained 100 µg 2,3-oxidosqualene (Sigma-Aldrich) and was added to a centrifuge tube containing Tris-HCl buffer (pH 6.0). Twenty micrograms of crude enzyme was added and incubated at 37 °C for 1 h. The reaction was stopped by heating at 100 °C [31]. The dammarenediol product was used for quantitative analysis by LC-MS. The chromatographic column was a C18 column (150 mm × 4.6 mm). The mobile phase was methanol:water (85:15, *v*/*v*); the flow rate was 1.0 mL/min; the column temperature was 30 °C, and the detection wavelength was 203 nm. The MS operating conditions were as follows: electrospray ionization source (ESI); all spectra were obtained in positive mode; declustering potential (DP) voltage: 70 V; and EP voltage: 30 V. The product amount of dammarenediol was calculated by comparing with the peak area ratio of the standard (1 mg/L).

#### 4.5.7. CYP450 Activity

The P450 activity was determined by the NADPH subtraction method. The NADPH oxidation assay determined the activity of CYP450 towards different substrates. The reaction mixture was a total volume of 1 mL and contained 0.3 mM tetradecanoic acid solution in DMSO (final concentration, 2%) and 20 µg crude enzyme in 50 mM potassium-phosphate buffer (pH 7.5). The reaction was initiated upon the addition of 100 µL of a 1.5 mM aqueous NADPH solution, followed at 340 nm (εNADPH = 6.22 mM^−1^·cm^−1^), and lasted 5 min [38].

#### 4.5.8. GT Enzyme Activity

The enzyme reaction system had a total volume of 0.5 mL and contained crude enzyme (20 µg), Tris-HCl buffer, 5.0 mM UDPG, and 0.5 mM ginsenoside Rh2, and was incubated for 30 min. Then, *n*-butanol (0.2 mL) was added to the reaction to stop the reaction after completion. The reactions were centrifuged at 12,000 r/min for 10 min, the *n*-butanol phase was taken, *n*-butanol was evaporated under reduced pressure at 45 °C, and the residue was dissolved into 0.05 mL of HPLC-grade methanol. Enzyme activity unit definition: 37 °C conditions, 1 min to produce 1 μmol Rg3, and the amount of enzyme required. Quantitative estimation of Rg3 production was by a standard curve of different concentrations of Rg3.

### 4.6. Statistical Analysis

Correlation analysis was conducted using SPSS software. The *p*-value was the probability and was used to compare the confidence coefficient among the groups of data using the paired *t*-test. If *p* < 0.05, this indicated that there was a significant correlation between groups of data. All results were displayed by R Core Team (2014).

## Supplementary Materials

Supplementary materials can be found at www.mdpi.com/1422-0067/18/12/2682/s1.
