# Supplementary material for: Study on the Correlation between Gene Expression and Enzyme Activity of Seven Key Enzymes and Ginsenoside Content in Ginseng in Over Time in Ji’an, China
Source: Int J Mol Sci. 2017 Dec 11;18(12):2682. doi: 10.3390/ijms18122682 (PMC5751284; doi:10.3390/ijms18122682)
Supplement: Supplementary file 1 [file ijms-18-02682-s001.pdf]

The expression of key enzymes

The primers of key genes were in Table S1. The gene expression of fresh ginseng from different regions was treated with the formula $2^{-\Delta\Delta Ct}$.

Table S1. The gene primers of the key enzymes

	Genebank ID	Forward primer	Reverse primer
HMGR	GQ455990	5'-ATGGTGTCCAAGGGTGTTCAG- 3'	5'-CTTTCCTCGCCCTTCAATCC-3'
FPS	DQ087959	5'-ACCCTGTTGGTTCAGATTACCTAAG-3'	5'-ATCCAACAGATCCACATAGTAAGGC-3'
SS	AB010148	5'-TTGGAAGCGGTTACCAGGAG-3'	5' -GAGGCATGGAAGAGCTTTGAC-3'
SE	AB122078	5'-CATTAGAACCATGCCAAACAGAA-3'	5' -TGGCGCATATTGAAAGCATC-3'
DS	GU183405	5'-CAGCGGAACGATTGACACTATTC-3'	5' -ATCGTGCTGTGCCCCTCAAT-3'
GT	KM491309	5'-GGCATCGCTTCTTGTGGTCC-3'	5' -GCCCATCCAATCACTTTTCC-3'
CYP450	AB122079	5'-AGATGCCGTTGTCGGAGTTG-3'	5'- CGCAACAGGGTGTAGCCTTA-3'

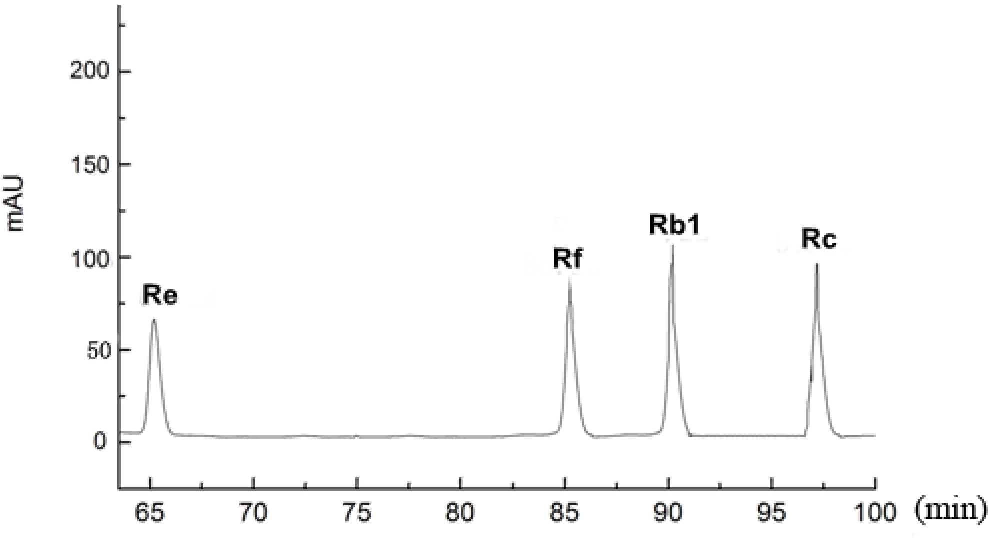

Figure S1. The retention time of different ginsenosides. (The mixture of standards of individual ginsenoside. Ginsenosides in the root were analyzed by HPLC and were separated at different time points).
